# A decade of molecular preimplantation genetic diagnosis of 350 blastomeres for beta-thalassemia combined with HLA typing, aneuploidy screening and sex selection in Iran

**DOI:** 10.1186/s12884-022-04660-9

**Published:** 2022-04-15

**Authors:** Yeganeh Keshvar, Solmaz Sabeghi, Zohreh Sharifi, Kiyana Sadat Fatemi, Panti Fouladi, Shahrzad Younesi Khah, Faezeh Rahiminejad, Atefeh Joudaki, Masoume Amini, Hamideh Bagherian, Marefat Ghaffari Novin, Mansoureh Movahedin, Marzieh Mojbafan, Sirous Zeinali

**Affiliations:** 1 Dr.Zeinali’s Medical Genetics Lab, Kawsar Human Genetics Research Center (KHGRC), Tehran, Iran; 2 Faculty of Advanced Sciences and Technology, Department of Genetics, Tehran Medical Sciences, Islamic Azad University, Tehran, Iran; 3 Biology and Anatomical Sciences Dept School of Medicine, ShahidBeheshti Uiversity of Medical Sciences, Tehran, Iran; 4 Aban IVF Center, Aban General Hospital, Tehran, Iran; 5 Anatomical Science Dept, Medical Science Faculty, TarbiatModares University, Tehran, Iran; 6 Gandhi IVF Center, Gandhi Hospital, Tehran, Iran; 7 Department of Medical Genetics and Molecular Biology, Faculty of Medicine, Iran University of Medical Sciences (IUMS), Tehran, Iran; 8 Department of Medical Genetics, Ali-Asghar Children’s Hospital, Iran University of Medical Sciences, Tehran, Iran

**Keywords:** Molecular PGD1, Nested PCR2, STR3, IVF4, Iran5

## Abstract

**Background:**

Preimplantation genetic diagnosis (PGD) has been developed to detect genetic disorders before pregnancy which is usually done on blastomeres biopsied from 8-cell stage embryos obtained from in vitro fertilization method (IVF).

Here we report molecular PGD results for diagnosing of beta thalassemia (beta-thal) which are usually accompanied with evaluating chromosomal aneuploidies, HLA typing and sex selection.

**Methods:**

In this study, haplotype analysis was performed using short tandem repeats (STRs) in a multiplex nested PCR and the causative mutation was detected by Sanger sequencing.

**Results:**

We have performed PGDs on 350 blastomeres from 55 carrier couples; 142 blastomeres for beta-thal only, 75 for beta-thal and HLA typing, 76 for beta-thal in combination with sex selection, and 57 for beta-thal and aneuploidy screening. 150 blastomeres were transferable, 15 pregnancies were happened, and 11 babies born.

We used 6 markers for beta-thal, 36 for aneuploidy screening, 32 for sex selection, and 35 for HLA typing. To our knowledge combining all these markers together and the number of STR markers are much more than any other studies which have ever done.

**Conclusions:**

PGD is a powerful diagnostic tool for carrier couples who desire to have a healthy child and wish to avoid medical abortion.

**Supplementary Information:**

The online version contains supplementary material available at 10.1186/s12884-022-04660-9.

## Background

Preimplantation genetic diagnosis (PGD) has become an available method for couples who are at risk of transmitting a known genetic disorder to their offspring and wish to avoid pregnancy termination. Because of religious and ethical issues, prenatal genetic diagnosis (PND) is not always acceptable; therefore, PGD could be an appropriate alternative. It can also be an important technique to improve the efficiency of in vitro fertilization (IVF) [1].

PGD is being performed by two main methods; amplification-based and fluorescence in situ hybridization-based (FISH). FISH is an older technique usually used for preimplantation genetic screening (PGS). FISH can be used for embryo gender selection, inherited diseases and chromosomal abnormalities [2].

In amplification-based method, molecular tests can be performed on blastomeres biopsied from 8-cell stage embryos and abnormalities in blastomeres are detectable before transferring embryos into the mother’s uterus. Molecular tests are usually performed using fragment analysis and haplotype mapping as well as Sanger sequencing [3]. In most cases sets of short tandem repeats (STRs) markers as well as mutated regions are amplified in a two-step multiplex reaction. The information provided will be used to draw haplotypes which aids in tracking the defective chromosome or gene. Therefore, Preimplantation Genetic Haplotyping (PGH) is utilized to determine segregation pattern of the mutated gene in the offspring.

Beta-thal is an autosomal recessive blood disorder which results in life-threatening anemia and affected individuals may require regular blood transfusion for survival [4]. Iran is one of the countries located on thalassemia belt with more than 13,000 registered major thalassemia patients [5]. Presently, there are more than two million carriers of beta-thal in Iran [6].

PGD is performed for many conditions such as monogenic disorders like beta-thal, aneuploidy screening, sex selection and HLA typing. PGD for aneuploidy screening will increase the rate of implantation; reduce risks of spontaneous abortion and chromosomally abnormal fetus. Using PGD can radically reduce the possibility of trisomy conceptions which may happen by raised maternal age [7].

PGD for sex selection can be used for medical purposes like preventing birth of affected children with X-linked disorders of an unknown gene. Another advantage of using PGD for sex selection is that it is also able to investigate aneuploidies related to sex chromosomes [8].

PGD has also been used for HLA typing which allows selection and transfer of unaffected embryos that have a closely matched-HLA with an existing affected child in the family. HLA-identical siblings provide the best chance to achieve a successful transplantation for the recipient. Therefore, if no HLA-identical donor is available in the family or HLA registries, an increasing number of couples with an affected child are considered for using PGD techniques for therapeutic purpose [9].

Here in this study, we present our ten-year experience on molecular PGD in 350 blastomeres of beta-thal carrier couples in accompany with other situations.

## Methods

### Subject

Fifty-five carrier couples who were candidate for PGD had been referred to Dr. Zeinali’s Medical Genetics Lab, Kawsar Human Genetics Research Centre (KHGRC) since 2009. Genetic counselling was performed and informed consent was obtained from all participant families. This study was approved by research committee of KHGRC and all methods were performed in accordance with the relevant guidelines and regulations.

### Molecular genetic analysis

Genomic DNA was extracted using salting out method [10]. STR markers were determined using Map viewer (http://www.ncbi.nlm.nih.gov/projects/mapview), SERV (http://www.igs.cnrs-mrs.fr/SERV/) and Tandem Repeat Finder (TRF) software (http://tandem.bu.edu/trf/trf.html).

We gave new name to the new STR markers; I, U and D stand for inside, upstream and downstream of the desired gene respectively. The numbers denote distance from the gene (e.g. 8.05 × 10^5^ base pairs).

Primers for STR analysis were fluorescently labeled by FAM, NED, VIC, and PET and were then used in a multiplex PCR set. Bioinformatics analysis of identified variants was performed using PolyPhen-2 (http://genetics.bwh.harvard.edu/pph2/) and SIFT (http://sift.bii.a-star.edu.sg/), Mutation Taster (http://www.mutationtaster.org/) and Human Splicing Finder (HSF) software. Variant interpretation was performed according to the American College of Medical Genetics and Genomics (ACMG).

Fragment analysis and sequencing were done using an ABI 3130XL Genetic Analyzer (Thermo Fisher Scientific, USA, and TF).

Six STR markers flanking beta-globin gene were used for beta-globing gene segregation [11]. 35 STR markers surrounding the HLA class I, II and III regions as well as its telomeric and centromeric regions on chromosome 6 were selected for human leukocyte antigen (HLA) typing. We deployed 32 STR markers for sex selection, 36 STR markers for detecting aneuploidies, and 5 STR markers for cell identification, in which the latter markers are located on different chromosomes and often applied for confirming paternity (Table S1, S2). Primers for amplifying these markers are available upon request.

### Embryo biopsy and cell lysis

The obtained zygotes were cultured for 72 h [12]. A hole was made on each embryo to be biopsied using an infrared (1480 nm) laser (Fertilase; Octax, Herbron, Germany). Single blastomere was removed from each embryo through the hole using a fine needle with internal diameter of 35 mm. Individual blastomere from each embryo was washed twice in fresh droplets of PBS, PH 7.4 [12]. Blastomeres were placed in a buffer containing 5.5 µl of cell lysis buffer (0.5 µl 10 × buffer, 0.5 µl tween 1%, 0.5 µl triton 1%, 0.5 µl proteinase K 20 mg/ml and 3.5 µl dH_2_O). Then they were lysed by incubation at 45^0^C for 15 min and then at 96^0^C for 20 min to complete lysis of the cells [9]. All the procedure has been carried out in a semi-clean room under Class II laminar flow.

### DNA amplification

Each STR marker was individually checked for each family for being informative and selected STR markers and exon/s containing the causative mutation/s were amplified in two consecutive reactions using nested PCR method [11].

### Embryo transfer

Transferable embryos were implanted into the mothers’ uterus in the IVF clinic. Unaffected or matched (i.e. for HLA cases) embryos were subsequently transferred and two weeks later, pregnancy tests were performed. If pregnancy was successful, PND was offered and families performed (with consent) at around 11^th^ weeks of gestation to confirm the PGD results.

## Result

Fifty five beta-thal carrier couples referred to our PGD center since 2009. In total we have performed molecular based PGD on 350 blastomeres in which 78 were normal, 129 were carrier, 85 were affected and 58 of them were without nucleus. 150 blastomeres were transferrable, others were either non-transferable or their implantations did not end up to successful pregnancies which is results from the failure of IVF technique. The rate of pregnancy in our study was 15 out of 55 couples (27%) in which 5 out of 15 pregnancies were aborted before 11 weeks of gestation, and the remaining 10 were successful. Table 1 summarizes the results of our PGD cases referred to beta-thal analysis only and in combination with other requested tests. We would include sequencing primer for a specific exon/s harbouring point mutation or small insertion or deletion. However, when the deletion or other complex rearrangements were involved, we would only use haplotyping to determine the fate of cell being analysed.

**Table 1:** Molecular Statistical findings of blastomeres studied in the present project

	Beta thalassemia only	Beta thalassemia + sex selection	Beta thalassemia + HLA typing	Beta thalassemia + Aneuploidy screening
Number of total blastomeres	142	76	75	57
Number of normal blastomeres	30	16	17	15
Number of carrier blastomeres	53	28	31	17
Number of affected blastomeres	36	18	15	16
Number of blastomeres without nucleus	23	14	12	9
Number of male blastomeres	-	25	-	-
Number of female blastomeres	-	28	-	-
Number of blastomers with chromosomal aberrations^a^	-	9	-	20
Number of full- matched HLA blastomeres^b^	-	-	14	-
Number of half- matched HLA blastomeres	-	-	27	-
Number of non-matched HLA blastomeres	-	-	22	-
Number of transferable blastomeres	83	38	10	19
Number of pregnancies	7	4	2	2
Number of miscarriages	2	1	-	2
Ongoing pregnancies	-	-	-	-
Number of babies born	5	2(twins) + 2	2	-

^a^The numerical chromosomal aberrations can be investigated in this study

^b^As some of the full matched blastomeres are also affected with beta thalassemia, they are undesirable candidates for transfer

### Beta thalassemia only

In order to perform PGD for beta-thal, several sets of STR markers were found and tested on at least 60 chromosomes to have preliminary allele frequency and heterogeneity. In each PGD cycle, only markers with high allele frequency were used to enable us to determine haplotype and segregation pattern. Finally 6 STRs were kept and made to become ready to be used in PGD and PND cases in our centre [11]. We had 22 couples who referred to our centre for detecting beta-thal only, and 7 pregnancies have been achieved in which five of them were successful and resulted in three carrier boys and two normal girls.

### Beta thalassemia and HLA typing

For HLA typing, more than 30 different STR markers have been selected to cover the whole HLA region as well its telomeric and centromeric regions. Allele frequencies and heterogeneities of these STR markers were tested as mentioned above. For HLA typing of embryos, we used at least 15 markers in which at least 3 markers covered the telomeric and 3 ones surrounded the centromeric ends of the cluster. These markers would act as safeguard for detecting possible crossovers.75 blastomeres belonging to12 couples were analyzed for beta-thal and HLA typing and 10 unaffected full-matched blastomeres were achieved. Two families were investigated for both beta-thal and HLA typing which resulted in two full HLA-matched beta- thal carrier babies.

### Beta thalassemia and sex selection

More than 30 STRs surrounding the human X and Y chromosomes have been chosen and tested for sex typing, but for PGD purpose about 10 out of 30 STR markers were used in the final primer mix since other primers for the beta-thal had to be used as well. Twelve couples requested for beta-thal combined with sex selection as it was revealed in Table 1. Four babies born from the selected embryos resulting in the birth of a normal boy, a carrier girl, and a twin birth (a carrier boy and girl).

### Beta thalassemia and aneuploidy screening

We have 36 STR markers for aneuploidy screening. In order to investigate cells for beta-thal and aneuploidy screening, almost four markers for each chromosome of 21, 18, 16, 13, X and Y would be used.

Nine couples were referred for assessing beta-thal and aneuploidy screening and 2 pregnancies were achieved.

We prefer to use some markers located on chromosomes 2, 4, 6, 7 in PGD cycle as an identifier of each cell or cell fingerprints regardless of family have asked for it or not. These markers which we call them human identification (HID) markers (Table S2) can help us to improve PGD outcome when embryos are transferred. For example, in a case of PGD referred for beta-thal only; in addition to the markers used for detecting beta-thal, we used HID markers such as D7S820, D2S338, SE33, and FGA for identifying each cell.

## Discussion

PGD has introduced a valuable and sometimes irreplaceable procedure and brings several technically challenging areas together like in vitro fertilization, embryo biopsy and culture, and molecular genetic diagnosis on a single cell. Using multiplex PCR in PGD which simultaneously amplify many linked informative polymorphic markers to a specific gene, was found to be quite helpful for the detection of both contamination and allele dropouts (ADOs) [13]. ADO is defined as the failure or preferential amplification of only one allele and is the primary cause of misdiagnosis in PGD especially if a single approach like mutation detection is applied [14]. Concurrent use of several polymorphic STR markers can help us to overcome misdiagnosis by reducing the error rate due to ADO from as high as 27% (in blastomeres for single cell) to almost 0% when several markers are used [15].

In addition to ADO, undetected crossovers between the wild type and mutant alleles is another leading cause of misdiagnosis in molecular PGDs. Crossover between markers and a specific variant; i.e. mutation cannot be completely ruled out even for very closely linked markers, therefore it is imperative to design markers likes STRs flanking a specific locus or variant [16].

In PGD for monogenic disorders, single cell PCR is extremely susceptible to contamination by extraneous DNA which can lead to misdiagnosis in DNA amplification-based PGDs. In this study, we tried to establish a reliable PGD test allowing simultaneous detection of recombination, contamination and ADOs using polymorphic STR markers [9]. In order to reduce contamination, two different areas were used for pre-PCR and post-PCR in our lab and even different staffs worked in these areas. All pre-PCR reactions were carried out in a semi-clean room under class II Laminar flow. To detect sources of possible contamination, DNA samples were prepared and STR profiles were generated from all PGD and IVF staffs [17]. Only in few early cases we could see some possible contaminations for one or more STR markers but we had never seen a complete failure of PGD due to external DNA contamination.

Using STR markers is very useful for performing PGDs in β-thal carrier couples specially when there is a time constraint to find the causative mutation and also it can be helpful in confirming direct mutation.

Although haplotype analysis is very practical, it faces some limitations like when we confront a family who has some un-informative markers or when we have limitations to access the family members for genotyping. Haplotype analysis is strongly depending on heterozygosity of markers [11].

In this study, we assessed 55 couples who had been referred to Dr. Zeinali’s Medical Genetics Lab, Kawsar Human Genetics Research Centre (KHGRC) for PGD of the beta-thal only or in combination with HLA typing, sex selection and/or aneuploidies screening. In total, 40.6% of blastomeres were assessed only for the beta-thal, and the remaining 59.4% were analysed simultaneously for the beta-thal and sex selection, HLA typing and aneuploidy screening. To our knowledge combining these markers all together and the number of STR markers to make several diagnosis in a single cell which we used in each PGD cycle are much more than any other studies that have ever done.

In the following paragraphs we are going to compare the present work with the other studies including ß‐thalassemia, HLA typing, sex selection, and aneuploidy screening: we must mention a study which was performed in Italy on 23 couples at risk of ß‐thalassemia and 150 embryos had been analysed. 98 embryos were unaffected and 75 out of them were transferred. In total four clinical pregnancies were obtained. There is no information about the number of born babies [18]. In our study, 83 (58.4%) blastomeres were unaffected (normal and carries) and transferable for the beta-thal only and 150 blastomeres were unaffected in conjugation with other situations (Table 1), which respectively resulted in 7 (8.4%) successful pregnancies and 5 (6%) born babies for the beta-thal only and 15 pregnancies (27%) and 9 born babies (16%) in total which was a significant result comparing with this study.

A PGD-HLA typing program carried out in Spain on 7 couples who provided a total of 202 embryos which used 20 STR markers surrounding the HLA locus. The rate of HLA-identical unaffected embryos was 8.4%, and the fertilization rate was 59.9% [19]. In the present study we had 35 potential STR markers for HLA typing which was used along with STR markers for the beta-thal simultaneously. 13.3% HLA-identical un-affected embryos from 12 families were transferred and 20% of transferable blastomeres were born. Comparing with the research done in Spain, we used much more STR markers simultaneously in a test tube to evaluate both the beta-thal and HLA typing.

In a study which had been performed in Belgium for checking sex and ploidy status of the embryos in 17 couples using fluorescent in-situ hybridization (FISH) technique, chromosomal abnormalities of X, Y and 18 was checked and five couples had successful pregnancies [20]. In another study by Malpani et al., 42 cycles for sex selection were performed using FISH technique and 106 embryos were transferred. Finally 16 biochemical and 14 clinical pregnancies were achieved resulting in nine live births with five ongoing pregnancies [21]. In our study, we detected combination of sex and the beta-thal in 12 couples in which 3 out of them had successful pregnancies (25%). Using STR markers in our study help us to assess both situations like sex typing and disorders such as beta-thal which are often results from point mutations but detecting such kind of disorders is not probable in PGD by FISH method which was used in mentioned studies above.

In a study which had been carried out in Thailand, beta-thal and Down's syndrome were analysed in two couples; they have provided 17 embryos which two out of them were found to be affected by Down’s syndrome and this strategy resulted in the first baby born following PGD for a single gene disorder in South East Asia in 2016 [22]. In our study assessing the beta-thal and aneuploidy status, we checked 16.3% of all blastomeres for this purpose, in which 33.3% of them were transferable. The pregnancy rate was 10.5% out of all transferrable blastomeres. PGD combined with PGS in this study revealed that 35% of all studied blastomeres had chromosomal aneuploidies.

Detection success rate was determined based on the PND test results. For all cases of successful PGD pregnancy, PND test were performed in the 11^th^ week of pregnancy: The embryos were checked for the thalassemia and other situations like HLA typing, sex selection, or aneuploidy screening, and all the fetuses were as same as it was molecularly diagnosed in the PGD cycle. Transplantation success rate which cause pregnancy was 27% which resulted in 11 born babies (Table 1). The remaining blastomres which did not end up to successful pregnancies were results from the failure of IVF. Mother's age, number of recovered oocytes, infertility of couples, number of transferred embryos and selecting an appropriate embryo to transfer are prominent factors affecting the success rate of IVF [23]. The optimal delivery rate is 26- to 30-year-old, which gradually decreased with maternal age [23]. In this study, we found a significant relationship (*p* < 0.05) between the maternal age and successful pregnancy. Of the 15 women who had successful pregnancies, 11 were under 30 years old (73.6%).

## Conclusions

In conclusion, PGD grants the opportunity to have unaffected children to high risk couples. This article not only summarizes the data of PGD technology and clinical application, but also offers the ability to detect 2 or even 3 conditions simultaneously on 10 embryos in one round of PGD cycle. For example, we were able to diagnose beta-thalassemia along with aneuploidy screening and sex selection (Fig S1).

This is a new point of view of our research that a large number of markers were examined and all these markers were combined together to make diagnoses of several situations on a single cell in each PGD cycle.

## Supplementary Information

**Additional file1: Fig S1. **A carrier couple for beta thalassemia who had also a carrier child and they were referred to our centre for the beta-thal, aneuploidy screening and sex selection. The haplotype represented a PGD result for 7 single cell. The STR markers were shown on left. Blastomeres 4 and 6 were affected by thalassemia. The second blastomere is an affected female with monosomy 13 and the last one is a carrier male who had trisomy 21. There are 3 transferable blastomeres (1, 3, and 5).

**Additional file 2:** **Table S1. **Characterization of aneuploidy screening and sex selection STRmarkers used in this study

**Additional file 3:** **TableS2.** Characterization of HLA and HID STR markers used in this study.

## Data Availability

The datasets generated and/or analysed during the current study are available in the published articles [11, 24–26] that are known STRs and were previously used, and the remaining STRs used in this article were designed by our center and deposited in the following web link: https://www.ncbi.nlm.nih.gov/probe/?term=Sharifi
