# Supplementary figures and images for: A decade of molecular preimplantation genetic diagnosis of 350 blastomeres for beta-thalassemia combined with HLA typing, aneuploidy screening and sex selection in Iran
Source: BMC Pregnancy Childbirth. 2022 Apr 15;22:330. doi: 10.1186/s12884-022-04660-9 (PMC9013130; doi:10.1186/s12884-022-04660-9)

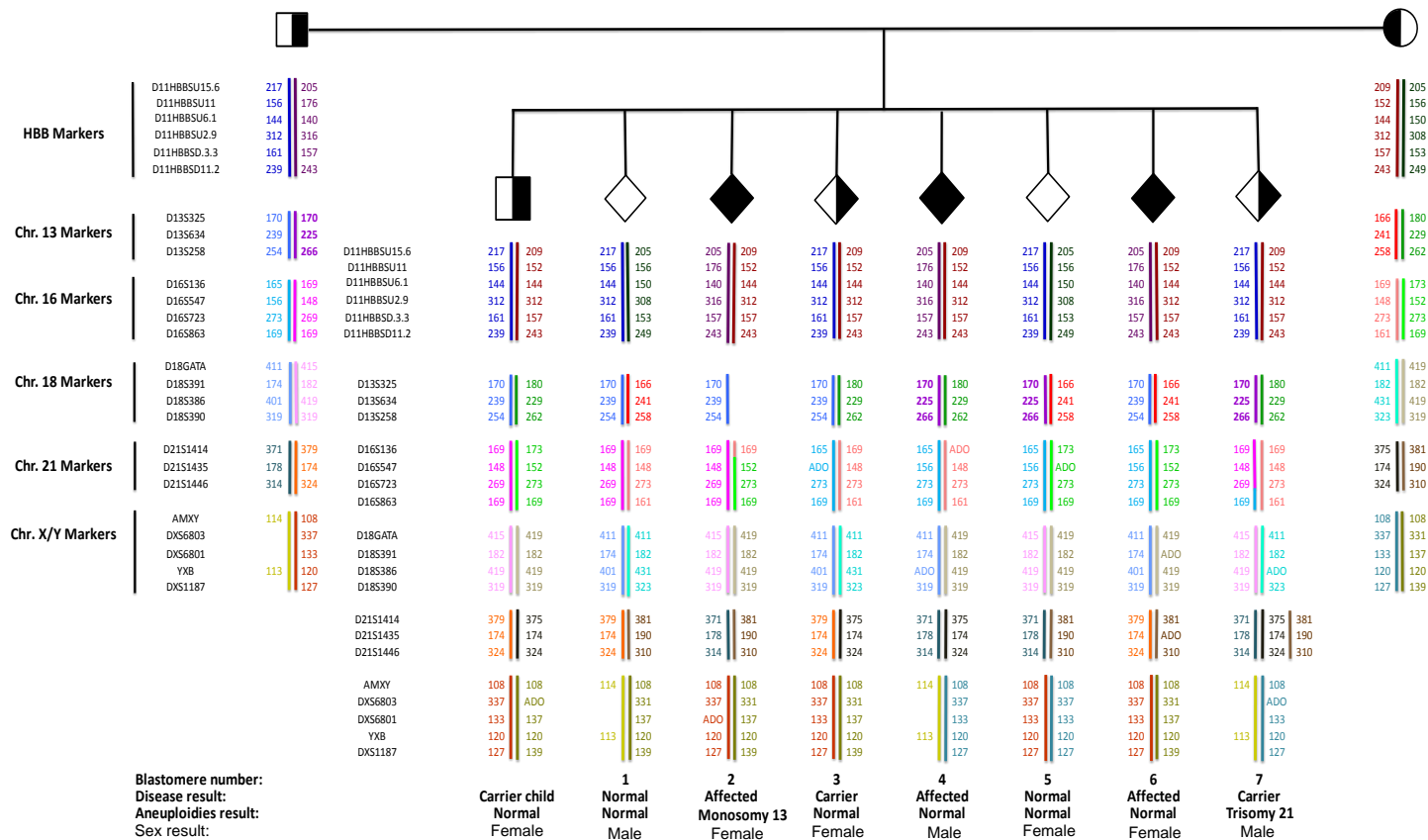

Supplement: Supplementary file 1 — Additional file1: Fig S1. A carrier couple for beta thalassemia who had also a carrier child and they were referred to our centre for the beta-thal, aneuploidy screening and sex selection. The haplotype represented a PGD result for 7 single cell. The STR markers were shown on left. Blastomeres 4 and 6 were affected by thalassemia. The second blastomere is an affected female with monosomy 13 and the last one is a carrier male who had trisomy 21. There are 3 transferable blastomeres (1, 3, and 5). [file 12884_2022_4660_MOESM1_ESM.pdf]
