# Supplementary material for: A decade of molecular preimplantation genetic diagnosis of 350 blastomeres for beta-thalassemia combined with HLA typing, aneuploidy screening and sex selection in Iran
Source: BMC Pregnancy Childbirth. 2022 Apr 15;22:330. doi: 10.1186/s12884-022-04660-9 (PMC9013130; doi:10.1186/s12884-022-04660-9)
Supplement: Supplementary file 2 — Additional file 2: Table S1. Characterization of aneuploidy screening and sex selection STRmarkers used in this study [file 12884_2022_4660_MOESM2_ESM.docx]

**Table S1:** Characterization of aneuploidy screening and sex selection STR markers used in this study.

| **Aneuploidy Screening**  **STR Markers** | **Marker** | **Size Range (bp)** | **Fluorescent Label** | **Location** |
| --- | --- | --- | --- | --- |
|  | D13S325 | (125-190) | FAM | 13q14.11 |
|  | D13S305 | (451-483) | VIC | 13q13.3 |
|  | D13S252 | (405-455) | FAM | 13q12.2 |
|  | D13S634 | (220-248) | VIC | 13q21.33 |
|  | D13S258 | (250-320) | VIC | 13q21.33 |
|  | D13S797 | (115-155) | PET | 13q33.2 |
|  | D16S252 | (238-298) | NED | 16p12.1 |
|  | D16S136 | (136-196) | VIC | 16p13.12 |
|  | D16S488 | (210-270) | VIC | 16q12.1 |
|  | D16S547 | (120-170) | NED | 16q12.2 |
|  | D16S723 | (243-300) | PET | 16q22.1 |
|  | D16S863 | (140-200) | FAM | 16q24.1 |
|  | D18S390 | (210-250) | FAM | 18q22.3 |
|  | D18S391 | (160-200) | VIC | 18p11.31 |
|  | D18S535 | (275-325) | PET | 18q12.3 |
|  | D18S386 | (376-444) | NED | 18q22.1 |
|  | GATA178F11 | (370-430) | PET | 18p11.32 |
|  | D21S1809 | (260-284) | FAM | 21q22.2 |
|  | D21S1446 | (285-328) | FAM | 21q22.3 |
|  | IFNAR | (350-402) | FAM | 21q22.1 |
|  | D21S1435 | (149-210) | NED | 21q21.3 |
|  | D21S1414 | (350-430) | VIC | 21q21.1 |
|  | D21S1442 | (190-260) | NED | 21q21.3 |
|  | D21S1411 | (346-460) | NED | 21q22.3 |
| **Sex Selection STR Markers** | AMXY^*^ | 108 (X)/ 114 (Y) | FAM | X p22.2/ Y p11.2 |
|  | DXS7132^*^ | (115-150) | VIC | Xq12 |
|  | SRY^*^ | 214(Y) (208-215) | VIC | Yp11.31 |
|  | Y/X B^*^ | 113 (Y)/ 120 (x) | NED | Y p11.2/ Xq21.31 |
|  | HPRT^*^ | (145-185) | NED | Xq26.3 |
|  | DXS6803^*^ | (315-344) | NED | Xq21.31 |
|  | DXS6801^*^ | (236-263) | PET | Xq21.32 |
|  | DXS1187^*^ | (130-187) | FAM | Xq26.2 |
|  | DYS437^*^ | (160-200) | PET | Yq11.21 |
|  | 7X^*^ | 215 (7)/ 238 (x) | PET | 7q34/ Xq13.3 |
|  | DX-TATC 13.3^*^ | (245-271) | PET | Xp21.2 |
|  | DXS981^*^ | (330-365) | PET | Xq13.1 |
|  | DXSF8SD1.6 | (332-392) | FAM | Xq28 |
|  | DXSF8SD15.9 | (164-224) | FAM | Xq28 |
|  | DXSF8SU6 | (276-336) | VIC | Xq28 |
|  | DXSF8SU6.1 | (248-308) | NED | Xq28 |
|  | DXSF8SD8.4 | (170-230) | PET | Xq28 |
|  | DXSF8SU8.7 | (349-409) | PET | Xq28 |
|  | DXSF8SD15.96 | (297-357) | VIC | Xq28 |
|  | DXSF8SD9.4 | (342-402) | NED | Xq28 |
|  | DXSF8SU2.4 | (199-259) | PET | Xq28 |
|  | DXDMDSI1 | (190-250) | PET | Xp21.2 |
|  | DXDMDSI2 | (183-243) | VIC | Xp21.2 |
|  | DXDMDSI3 | (186-246) | FAM | Xp21.2 |
|  | DXDMDSD13.3 | (260-320) | FAM | Xp21.2 |
|  | DXDMDSU1.6 | (181-241) | NED | Xp21.2 |
|  | DXDMDSU20.5 | (219-279) | PET | Xp21.2 |
|  | DXDMDSD17.9 | (204-264) | VIC | Xp21.2 |
|  | DXDMDSD15.7 | (166-226) | NED | Xp21.2 |
|  | DXDMDSU21.7 | (223-283) | NED | Xp21.2 |
|  | DXDMDSU6 | (125-185) | VIC | Xp21.2 |
|  | DXDMDSU6.9 | (149-209) | PET | Xp21.2 |

^*^ These markers are used in both aneuploidy screening and sex selection.
