# Supplementary material for: A decade of molecular preimplantation genetic diagnosis of 350 blastomeres for beta-thalassemia combined with HLA typing, aneuploidy screening and sex selection in Iran
Source: BMC Pregnancy Childbirth. 2022 Apr 15;22:330. doi: 10.1186/s12884-022-04660-9 (PMC9013130; doi:10.1186/s12884-022-04660-9)
Supplement: Supplementary file 3 — Additional file 3: TableS2. Characterization of HLA and HID STR markers used in this study. [file 12884_2022_4660_MOESM3_ESM.docx]

**Table S2:** Characterization of HLA and HID STR markers used in this study.

| **Location** | **Fluorescent Label** | **Size Range (bp)** | **Marker** | **HLA STR Markers** |
| --- | --- | --- | --- | --- |
| 6p22.3 | PET | (165-225) | D6HLATS232 |  |
| 6p22.3 | NED | (165-225) | D6HLATS237 |  |
| 6p22.3 | FAM | (202-262) | D6HLATS243 |  |
| 6p22.3 | NED | (435-495) | D6HLATS246 |  |
| 6p22.3 | PET | (258-318) | D6HLATS250 |  |
| 6p22.2 | VIC | (198-258) | D6HLATS252 |  |
| 6p22.2 | PET | (386-446) | D6HLATS259 |  |
| 6p22.2 | NED | (395-455) | D6HLATS262 |  |
| 6p22.2 | VIC | (211-271) | D6HLATS265 |  |
| 6p22.1 | PET | (177-237) | D6HLATS273 |  |
| 6p22.1 | VIC | (254-314) | D6HLATS278 |  |
| 6p22.1 | VIC | (396-456) | D6HLA1S281 |  |
| 6p22.1 | NED | (184-244) | D6HLA1S290 |  |
| 6p22.1 | FAM | (174-234) | D6HLA1S296 |  |
| 6p22.1 | NED | (299-359) | D6HLA1S301 |  |
| 6p21.33 | PET | (374-434) | D6HLA1S312 |  |
| 6p21.33 | NED | (208-268) | D6HLA3S320 |  |
| 6p21.32 | FAM | (267-327) | D6HLA3S321 |  |
| 6p21.32 | PET | (157-217) | D6HLA2S328 |  |
| 6p21.32 | PET | (434-494) | D6HLA2S331 |  |
| 6p21.32 | PET | (129-189) | D6HLA2S334 |  |
| 6p21.32 | PET | (313-373) | D6HLA2S334.5 |  |
| 6p21.31 | FAM | (253-313) | D6HLA2S339.8 |  |
| 6p21.31 | FAM | (156-216) | D6HLA2S344 |  |
| 6p21.31 | NED | (239-299) | D6HLA2S346 |  |
| 6p21.31 | FAM | (161-221) | D6HLACS359 |  |
| 6p21.31 | FAM | (362-422) | D6HLACS362.4 |  |
| 6p21.2 | FAM | (344-404) | D6HLACS366.3 |  |
| 6p21.2 | FAM | (149-209) | D6HLACS366.9 |  |
| 6p21.2 | PET | (172-232) | D6HLACS380 |  |
| 6p21.2 | NED | (176-236) | D6HLACS383 |  |
| 6p21.2 | PET | (209-269) | D6HLACS402 |  |
| 6p21.2 | FAM | (342-402) | D6HLACS404 |  |
| 6p21.1 | VIC | (143-203) | D6HLACS407 |  |
| 6p21.1 | FAM | (230-290) | D6HLACS415 |  |
| 16q24.1 | PET | (104-153) | D16S539 | **HID STR Markers** |
| 7q21.11 | FAM | (143-179) | D7S820 |  |
| 2q35 | VIC | (245-313) | D2S338 |  |
| 6q15 | NED | (251-311) | SE33 |  |
| 4q31.3 | PET | (195-255 ) | FGA |  |
